# Properdin: A Novel Target for Neuroprotection in Neonatal Hypoxic-Ischemic Brain Injury

**DOI:** 10.3389/fimmu.2019.02610

**Published:** 2019-11-29

**Authors:** Claudia Sisa, Qudsiyah Agha-Shah, Balpreet Sanghera, Ariela Carno, Cordula Stover, Mariya Hristova

**Affiliations:** ^1^Perinatal Brain Repair Group, UCL Institute for Women's Health, Maternal & Fetal Medicine, London, United Kingdom; ^2^Department of Infection, Immunity and Inflammation, University of Leicester, Leicester, United Kingdom

**Keywords:** properdin, complement, alternative pathway, hypoxia, ischemia, neuroprotection, neonate, infection

## Abstract

**Background:** Hypoxic-ischemic (HI) encephalopathy is a major cause of neonatal mortality and morbidity, with a global incidence of 3 per 1,000 live births. Intrauterine or perinatal complications, including maternal infection, constitute a major risk for the development of neonatal HI brain damage. During HI, inflammatory response and oxidative stress occur, causing subsequent cell death. The presence of an infection sensitizes the neonatal brain, making it more vulnerable to the HI damage. Currently, therapeutic hypothermia is the only clinically approved treatment available for HI encephalopathy, however it is only partially effective in HI alone and its application in infection-sensitized HI is debatable. Therefore, there is an unmet clinical need for the development of novel therapeutic interventions for the treatment of HI. Such an alternative is targeting the complement system. Properdin, which is involved in stabilization of the alternative pathway convertases, is the only known positive regulator of alternative complement activation. Absence of the classical pathway in the neonatal HI brain is neuroprotective. However, there is a paucity of data on the participation of the alternative pathway and in particular the role of properdin in HI brain damage.

**Objectives:** Our study aimed to validate the effect of global properdin deletion in two mouse models: HI alone and LPS-sensitized HI, thus addressing two different clinical scenarios.

**Results:** Our results indicate that global properdin deletion in a Rice-Vannucci model of neonatal HI and LPS-sensitized HI brain damage, in the short term, clearly reduced forebrain cell death and microglial activation, as well as tissue loss. In HI alone, deletion of properdin reduced TUNEL+ cell death and microglial post-HI response at 48 h post insult. Under the conditions of LPS-sensitized HI, properdin deletion diminished TUNEL+ cell death, tissue loss and microglial activation at 48 h post-HI.

**Conclusion:** Overall, our data suggests a critical role for properdin, and possibly also a contribution in neonatal HI alone and in infection-sensitized HI brain damage. Thus, properdin can be considered a novel target for treatment of neonatal HI brain damage.

## Introduction

Oxygen deprivation around the time of birth is a major cause of neonatal hypoxic ischemic (HI) brain damage affecting 1–3 per 1,000 live births in developed countries and increasing to 26 per 1,000 in the developing world (1). Of the affected neonates, 15–25% die during the neonatal period and 25% of the survivors develop neurological sequelae such as epilepsy, cerebral palsy and cognitive defects (2), resulting in significant psychological and socioeconomic burden on the patient, family and healthcare system (1).

The pathophysiology of HI brain damage involves inflammation, oxidative stress, excito-toxicity and cell death (3–6).

Pre-exposure of the preterm infant to a bacterial infection sensitizes the brain, making it more susceptible to the HI insult. Bacterial lipopolysaccharide (LPS)—the major component of the outer membrane of most Gram-negative bacteria—is a strong immune stimulator and enhances cerebral damage and lesions in HI brain injury (7, 8).

Therapeutic hypothermia (TH) is the standard clinical care for moderate to severe HI injury, however it is effective in only 55% of cases, while the remaining 45% of treated infants still develop neurological deficits (9). Thus, further studies on improving the success of TH and finding therapeutic alternatives are urgently required.

The effect of TH in infection-sensitized HI conditions is pathogen dependent (10). In a rat model of LPS-sensitized HI, TH failed to reduce mortality and tissue damage (11). In clinical studies looking at the effect of TH on neonates exposed to intrauterine infection, TH treatment did not counteract inflammation (12).

The lack of effect of TH in LPS-sensitized HI could be attributed to inter-individual variability (7). Additionally, body cooling following HI alone is suggested to be immunosuppressive (13, 14), therefore counteracting the physiological attempt of the immune system in fighting bacterial infection.

The inability of TH to protect the neonatal brain in LPS-sensitized HI, and its limited outcome in treatment of HI alone, support the urge to investigate new therapeutic alternatives or augmentation strategies for TH.

Such an alternative is targeting the complement system, a cascade of over 30 proteins critically involved in innate immunity. The activated complement promotes inflammation and anaphylatoxin release and comprises three pathways—classical, lectin, and alternative. While the classical pathway (CP) is mainly activated by external pathogens, the alternative one (AP) is spontaneously active and also amplifies the other two pathways (15). Properdin is a plasma glycoprotein released mainly by leukocytes in response to pro-inflammatory stimuli (16). It is the only known positive regulator of the AP; in fact, properdin facilitates the constitutively active AP either by stabilizing the C3 convertase C3bBb or by binding to susceptible surfaces, thus serving as a platform for *de novo* C3bBb assembly (17, 18). This causes opsonization of target molecules through C3b and further activation of the complement cascade, culminating in the formation of the membrane attack complex (C5b-C9).

Clinical data associates neonatal HI with depleted C3 expression (19) and increased serum levels of C3a and C5a following fetal acidosis (20). While the role of properdin in inflammation has been widely studied (21, 22), there is a paucity of data surrounding the role of properdin in neonatal HI. It could be speculated that HI upregulates properdin levels and leads to increased anaphylatoxin production and pro-inflammatory activation of microglia and astrocytes. This study aims to elucidate the role of properdin in neonatal HI alone and in LPS-sensitized HI in the short term. Our data demonstrate the neuroprotective effect of global properdin deletion in both HI alone and LPS-sensitized HI at 48 h post-HI, suggesting this complement regulator as an attractive therapeutic target in neonatal HI and LPS-sensitized HI.

## Materials and Methods

### Animal Use

Properdin-deficient mice were generated by site-specific genetic engineering, rendering mice deficient of the serum protein properdin and thereby lacking the amplification loop of complement activation (23). They have been maintained by crossing heterozygous properdin deficient female mice with wild type male C57Bl/6 mice and were obtained from the University of Leicester. Genotyping was performed on animals after treatment.

All animal experiments and care protocols were approved by the Home Office (PPL70/8784) and UCL Animal Welfare and Ethical Review Body. All procedures were carried out in accordance with the UK Animals (Scientific Procedures) Act 1986 and the ARRIVE guidelines. All experiments involved postnatal day 7 mice (P7) bred in house. At P7, the neonatal mouse brain development is comparable to a mid-third-trimester human fetus or newborn infant, with complete cortical neuronal layering, an involuted germinal matrix, and slightly myelinated white matter (24). Although slightly preterm, the murine P7 model of HI presents phenotypical similarities to the gray and white matter injury observed in humans, including tissue loss, cell-death, microglia-mediated immune response and astrogliosis as well as changes in neurological behavior (24).

Because properdin is located on the X-chromosome, mating of heterozygous properdin deficient females with wild type males yields male hemizygous, properdin-deficient and wild type mice (as well as female heterozygous and wild type mice). Therefore, only male pups were used in the experiments and were ideally controlled as littermates. According to clinical and experimental evidence, male mice may express a worse phenotype post-HI than female mice, with increased loss of male hippocampal volume after chronic postnatal hypoxia (25). All the assessments were performed blindly to the genotype.

### HI Insult

The surgical procedures, a variation of the Rice-Vannucci rodent HI model, were performed as previously described (7, 26–30). Briefly, a total of 30 P7 male mice, both wild type (*n* = 15) and with global properdin deletion (*n* = 15), were anesthetized using isoflurane (5% induction, 1.5% maintenance). The left carotid artery was permanently occluded (8/0 propylene suture) and the wound was closed with tissue glue. The mice were left to recover at 36°C and returned to the dam for 2 h. They were then moved to a hypoxia chamber and exposed to humidified 8% oxygen, 92% nitrogen (3 L/min) for 60 min (HI alone) or 30 min (LPS-sensitized HI) at 36°C (27), resulting in moderate to severe brain damage (27, 29, 30). In the infection-sensitized HI insult, 55 P6 pups from both genotypes were injected with *E. coli* lipopolysaccharide (LPS; 0.6 μg/g, serotype 055:B5; Fluka, UK) (*n* = 13 WT, *n* = 13 KO) or saline 12 h prior to surgery (*n* = 15 WT, *n* = 15 KO) (7, 29). The contralateral side of the brain served as an intra-animal control reference for ipsilateral damage.

### Tissue Sample Preparation

The animals were sacrificed at 48 h following the HI insult using intraperitoneally delivered pentobarbitone. They were perfused with 30 mL 4% paraformaldehyde (PFA) in phosphate-buffered saline (PBS). The brains were then extracted, post-fixed for 1 h in 4% PFA/0.1M phosphate buffer (PB) at 4°C, before being cryoprotected in 30% sucrose/PB solution for 24 h. The cerebellum was removed from each brain. The forebrains were frozen on dry ice, cut into 50 sequential 40 μm coronal sections starting from the fusion of corpus callosum, and the slices were stored at −80°C (7, 27, 29, 30).

### Immunohistochemistry and Histological Analysis

Five sections from each brain (400 μm apart) were rehydrated in distilled water and stained using immunohistochemistry as previously described (7, 27, 29, 30). Briefly, the sections were incubated overnight with rat anti-CD11b αM integrin subunit (1:5,000, Serotec, UK) or rabbit polyclonal anti-glial fibrillary acidic protein (GFAP) (1:6,000, DAKO, UK), primary antibodies, for 1 h with biotinylated goat anti-rat or -rabbit (1:100, Vector, UK) secondary antibodies, followed by incubation with Avidin-Biotinylated horseradish peroxidase Complex (Vector, UK) and visualization with diaminobenzidine/H_2_O_2_ (Fisher Scientific, UK) (7, 27, 29, 30).

Five further sections from each brain with the same spacing were stained using Terminal transferase mediated d-UTP nick end labeling (TUNEL) (Roche, UK). The staining procedure followed the manufacturer protocol with Co/Ni enhancement (7, 27, 29, 30).

Five more sections per brain with the same spacing were stained with Cresyl-Violet (Nissl).

### AlphaM Score

Assessment for αM integrin immunoreactivity as a marker for early microglial activation (7, 26–30) was performed as previously described (7, 27, 30). Two independent observers blinded to the genotype and treatment of the groups allocated semi-quantitative scores to each brain region, i.e., cortex, pyriform cortex, hippocampus, striatum, thalamus, and external capsule.

### Optical Luminosity

The central cytoskeletal framework of astroglia comprises GFAP, a type III intermediate filament found only in glial cells in the CNS. GFAP upregulation is seen during HI-triggered reactive gliosis (31). In order to quantify the intensity of the GFAP staining, we used optical luminosity values (OLV) as a well-established technique (7, 26–30). Images for ipsilateral and contralateral sides were captured with a Sony AVT-Horn 3CCD color video camera (24 bit RGB, 760 × 570 pixel resolution) in three different optical fields in cortex, pyriform cortex, hippocampus, striatum, thalamus and external capsule. We used Optimas 6.5 software to obtain the mean and standard deviation (SD) for OLVs. SD was subtracted from the mean for each image, and the resulting value was subtracted from the values acquired for the surrounding glass.

### TUNEL Assessment

As a measure of cell death at 48 h post-HI, the number of TUNEL + cells was counted in three different optical fields at ×20 magnification. The cortex, pyriform cortex, hippocampus, striatum, thalamus and external capsule were assessed. The counts were averaged per animal and per group.

### Infarct Volume Measurement

Cresyl violet dye stains Nissl bodies present in the cytoplasm of neurons. In this study we used Nissl stain to measure tissue loss in the cortex, pyriform cortex, hippocampus, striatum and thalamus. Nissl-stained brain sections were imaged with Sony AVT-Horn 3CCD color video camera (24 bit RGB, 760 × 570 pixel resolution) at ×1 magnification. The images were imported in Fiji Image J (NIH, USA), and the areas of intact staining in all 6 regions were outlined and bilaterally measured. The percentage tissue loss was then calculated by converting the measured uninjured areas into square millimeters and then transformed to a volume through multiplication by 400 μm. The sum of these volumes was then used to calculate the percentage of surviving brain tissue as ipsilateral/contralateral × 100 (32).

### Statistics

GraphPad Prism 8 (La Jolla, CA, United States) and SPSS 25.0 (IBM, USA) were used to perform all statistical analyses. The same six forebrain regions (cortex, pyriform cortex, hippocampus, striatum, thalamus, external capsule) were used for each outcome and each assay.

As the data from the Rice-Vannucci model are mostly non-normally distributed, we performed non-parametric Mann-Whitney test (HI alone set of experiments) to compare the effect of global properdin deletion in each brain region separately, and a two-tailed *p*-value was assumed. As the number of groups in the LPS-sensitized HI set of experiments was larger than two, we performed the non-parametric Kruskal-Wallis test followed by Bonferroni-corrected pairwise-contrasts. Alpha was set to 0.05. All data are graphically presented as Median ± IQR (interquartile range, presented as error bars). All hypotheses were two-tailed and all data illustrate the response in the ipsilateral (experimental) hemisphere. In our data, a main effect is the effect of an independent variable (treatment) on a dependent variable (damage marker) averaged across the levels of any other independent variables (brain regions).

## Results

### Global Properdin Deletion Reduces Cell Death and Microglial Activation Following Neonatal HI-Insult

**Figure 1:**
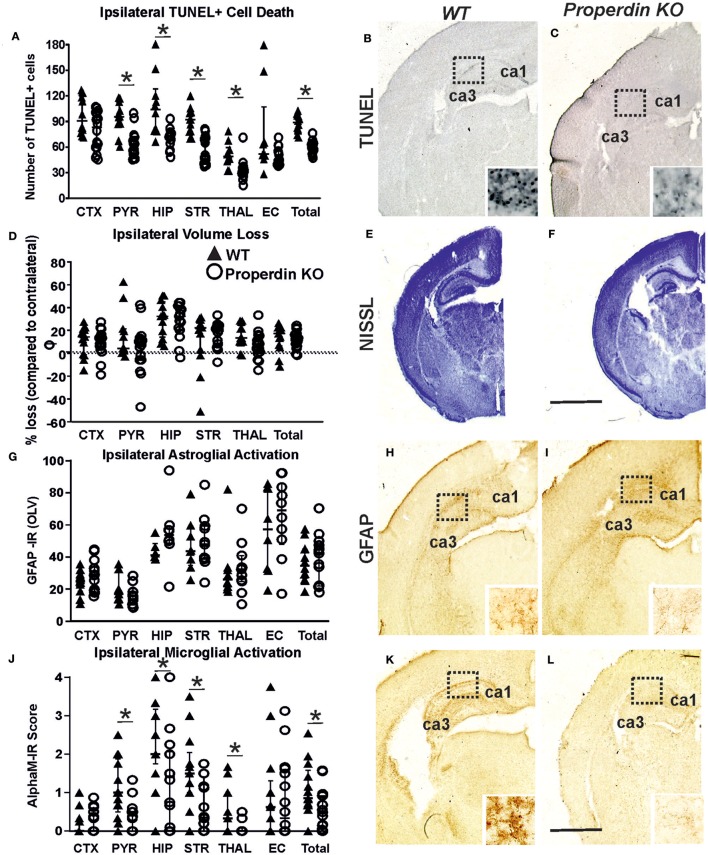
Global deletion of properdin in P7 mice significantly reduces cell death and microglial response at 48 h post-HI. **(A–C)** TUNEL+ staining of dying brain cells with fragmented DNA—Quantification **(A)** (number of TUNEL+ cells per 20× eye-field, Median ± IQR) and histochemical overview of the ipsilateral forebrain in wild type control **(B)** and animals with global properdin deletion **(C)**. Note the typical pyknotic nuclear morphology of the TUNEL+ cells, as well as their high density observed in the controls (**B**—insert, hippocampus) compared to the reduced number of such cells in the properdin KO group (**C**—insert, hippocampus). Compared to wild type controls, properdin deletion resulted in reduced TUNEL+ cell death across all 6 examined forebrain regions, with significant, individual decrease (Mann-Whitney test) in the pyriform cortex (*p* = 0.0004), hippocampus (*p* = 0.0008), striatum (*p* < 0.0001), thalamus (*p* = 0.0034) and overall (*p* < 0.0001). **(D–F)** Ipsilateral forebrain Nissl staining (Cresyl-Violet, at rostral parietal level)—Quantification of percentage of ipsilateral brain tissue volume loss (**D**, Median ± IQR) of wild type control **(E)** and properdin KO **(F)** animals. Compared to wild type controls global properdin deletion resulted in slight decrease of volume loss following neonatal HI, however the data did not reach significant values. **(G–I)** GFAP immunoreactivity—Quantification of ipsilateral reactive astrogliosis **(G)** in optical luminosity values (OLV, Median ± IQR), and low magnification ipsilateral overview in wild type control **(H)** and animals with global properdin deletion **(I)**. The inserts in H and I show higher magnification of the dotted regions in hippocampus. Global properdin deletion did not have an effect on astroglial activation following neonatal HI. **(J–L)** Activation of αM+ microglia—Ipsilateral αM microglial activation score (**J**, Median ± IQR) and low magnification ipsilateral overview in wild type control **(K)** and animals with global properdin deletion **(L)**. Note the strong microglial activation in the control wild type group with αM+ cells showing phagocytic morphology at high magnification (**K**—insert, hippocampus), compared to the properdin KO brains exhibiting a ramified phenotype (**L**—insert). Global properdin deletion reduced αM+ microglial activation across all 6 examined forebrain regions apart from cortex, with significant, individual decrease (Mann-Whitney test) in pyriform cortex (*p* = 0.008), hippocampus (*p* = 0.05), striatum (*p* = 0.02), thalamus (*p* = 0.04), and overall (*p* = 0.01). Wild type (*n* = 14) and global properdin deletion (*n* = 16) in all assessments. (*p < 0.05). CTX, cerebral cortex; PYR, pyriform cortex; HIP, hippocampus; STR, striatum; THAL, thalamus; EC, external capsule. Scale bars: **(E,F)** = 1,200 μm; **(B,C,H,I,K,L)** = 600 μm. inserts = 30 μm.

Global deletion of properdin significantly reduced brain damage markers (cell death and microglial activation) compared to wild type control animals at 48 h post-HI. As shown in Figures 1A–C, global properdin deletion significantly reduced the number of TUNEL+ cells compared to wild type controls, with individual significant decrease of 20–38% in pyriform cortex, hippocampus, striatum, thalamus and overall (*p* < 0.05, Mann-Whitney test). The TUNEL+ cells displayed the typical pyknotic nuclear morphology and high density in the control group (Figure 1B—insert, ipsilateral hippocampus) compared to the reduced number of such cells in the properdin KO brains (Figure 1C—insert, ipsilateral hippocampus).

The regional assessment presented in Figure 1D revealed slight decrease of ipsilateral brain tissue volume loss in the pyriform cortex and thalamus in the global properdin deletion group (Figure 1F) compared to wild type controls (Figure 1E), however the data did not reach significant values.

Assessment of ipsilateral astrogliosis through GFAP immunoreactivity (Figure 1G) showed that compared to wild type controls (Figure 1H), global properdin deletion had no effect on reactive astrogliosis (Figure 1I) following neonatal HI.

In addition to cell death, global properdin deletion had a significant effect on ipsilateral microglia activation score (Figure 1J) based on αM integrin immunoreactivity (Figures 1K,L). Regional assessment shown in Figure 1J revealed a reduction in activation score in the properdin KO group, with individual decrease of 21–76% in pyriform cortex, hippocampus, striatum, thalamus and overall (*p* < 0.05, Mann-Whitney test). At high magnification, the αM+ cells in the wild type control group showed phagocytic morphology (Figure 1K—insert, ipsilateral hippocampus) compared to the ramified phenotype of these cells observed in the animals with global properdin deletion (Figure 1L—insert, ipsilateral hippocampus).

### Global Properdin Deletion Reduces Brain Damage Following LPS-Sensitized Neonatal HI-Insult

**Figure 2:**
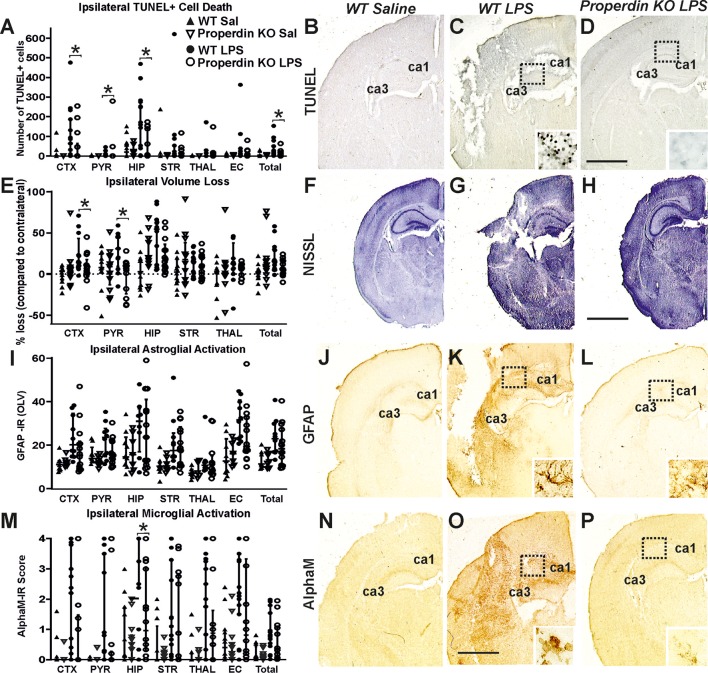
Global deletion of properdin in P7 mice significantly reduces cell death tissue loss and microglial response at 48 h post LPS-sensitized HI. **(A–D)** TUNEL+ staining of dying brain cells with fragmented DNA—Quantification **(A)** (number of TUNEL+ cells per 20× eye-field, Median ± IQR) and histochemical overview of the ipsilateral forebrain in saline-treated wild type **(B)**, LPS-treated wild type controls **(C)** and LPS-treated animals with global properdin deletion **(D)**. The saline-treated wild type animals showed a very low number of TUNEL+ cells **(B)**. Note the typical pyknotic nuclear morphology of the TUNEL+ cells as well as their high density observed in the LPS-treated wild type controls (**C**—insert, hippocampus) compared to the reduced number of such cells in the LPS-treated properdin KO group (**D**—insert, hippocampus). Compared to the wild type controls, properdin deletion resulted in reduced TUNEL+ cell death with a significant main effect (Kruskal-Wallis test, *p* = 0.007), and significant, individual decrease (Bonferroni correction) in cortex (*p* = 0.04), pyriform cortex (*p* = 0.03), hippocampus (*p* = 0.02) and overall (*p* = 0.05). **(E–H)** Ipsilateral forebrain Nissl staining (Cresyl-Violet, at rostral parietal level, Median ± IQR)—Quantification of ipsilateral brain tissue volume loss **(E)** of saline-treated wild type **(F)**, LPS-treated wild type controls **(G)**, and LPS-treated animals with global properdin deletion **(H)**. The saline-treated wild type animals showed very low levels of ipsilateral tissue loss **(F)**. Compared to wild type controls, global properdin deletion resulted in a decrease of volume loss following LPS-sensitized neonatal HI with a significant main effect (Kruskal-Wallis test, *p* = 0.0001) and significant individual decrease (Bonferroni correction) in cortex (*p* = 0.044) and pyriform cortex (*p* = 0.009). **(I–L)** GFAP immunoreactivity—Quantification of ipsilateral reactive astrogliosis **(G)** in optical luminosity values (OLV, Median ± IQR) and low magnification ipsilateral overview in saline-treated wild type **(J)**, LPS-treated wild type controls **(K)** and LPS-treated animals with global properdin deletion **(L)**. The saline-treated wild type animals showed very low levels of ipsilateral GFAP immunoreactivity **(J)**. The inserts **(K,L)** show higher magnification of the dotted regions in hippocampus. Compared to wild type controls, global properdin deletion decreased reactive astrogliosis with a significant main effect (Kruskal-Wallis test, *p* = 0.0001), however the sub-regional differences did not reach significant values. **(M–P)** Activation of αM+ microglia—Ipsilateral αM microglial activation score (**M**, Median ± IQR) and low magnification ipsilateral overview in saline-treated wild type **(N)**, LPS-treated wild type controls **(O)**, and LPS-treated animals with global properdin deletion **(P)**. The saline-treated wild type controls showed very low levels of αM+ microglia **(N)**. Note the strong microglial activation in the LPS-treated wild type control group with αM+ cells showing phagocytic morphology at high magnification (**O**—insert, hippocampus), compared to the LPS-treated properdin KO brains exhibiting a ramified phenotype (**P**—insert, hippocampus). Global properdin deletion reduced αM+ microglial activation with a significant main effect (Kruskal-Wallis test, *p* = 0.0001) and significant, individual decrease (Bonferroni correction) in hippocampus (*p* = 0.05). Saline wild type (*n* = 12), saline properdin KO (*n* = 14), LPS-treated wild type (*n* = 15) and LPS-treated properdin KO (*n* = 14) in all assessments. (*p < 0.05). CTX, cerebral cortex; PYR, pyriform cortex; HIP, hippocampus; STR, striatum; THAL, thalamus; EC, external capsule. Scale bars: **(F–H)** = 1,200 μm; **(B–D,J–L,N,O)**, *p* = 600 μm. inserts = 30 μm.

Global deletion of properdin significantly reduced brain damage markers (cell death, tissue loss and microglial activation) compared to wild type control animals at 48 h post LPS-sensitized neonatal HI. As shown in Figures 2A–D, global properdin deletion significantly reduced the number of TUNEL+ cells compared to LPS-treated wild type controls (main effect, *p* < 0.05, Kruskal-Wallis test), with individual decrease of 50–76% in all 6 regions, but reaching significance in cortex, pyriform cortex, hippocampus and overall (Bonferroni correction, *p* < 0.05). The saline-treated wild type controls showed very low number of TUNEL+ cells (Figures 2A,B), and global properdin deletion did not affect those numbers (Figure 2A). LPS-sensitization resulted in a substantial increase of TUNEL+ cell death observed in the wild type LPS-treated group (Figure 2C) compared to saline treated wild types (Figure 2B). The TUNEL+ cells displayed the typical pyknotic nuclear morphology and high density in the LPS-treated wild type group (Figure 1C—insert, ipsilateral hippocampus) compared to the reduced number of such cells in the LPS-treated properdin KO brains (Figure 1D—insert, ipsilateral hippocampus).

Regional assessment presented in Figure 2E revealed very low levels of ipsilateral tissue loss in the saline-treated wild type animals (Figure 2F), and global properdin deletion did not affect these levels (Figure 2E). LPS-sensitization resulted in an extensive increase of tissue loss observed in the LPS-treated wild type group (Figure 2G) compared to the saline treated wild type controls (Figure 2F). We observed an overall trend toward reduction of ipsilateral brain tissue volume loss of 13–66% across all 6 forebrain regions in the LPS-treated global properdin deletion group (Figure 2H) compared to LPS-treated wild type controls (Figure 2G) (main effect, *p* < 0.05, Kruskal-Wallis test), however the data reached significant values only in cortex and pyriform cortex (Bonferroni correction, *p* < 0.05).

Similarly, regional assessment of ipsilateral astrogliosis through GFAP immunoreactivity (Figure 2I) showed very low levels of reactive astrogliosis in saline-treated wild type controls (Figure 2J), and global properdin deletion did not affect those levels (Figure 2I). LPS-sensitization resulted in a considerable increase of astroglial activation observed in the LPS-treated wild type group (Figure 2K) compared to the saline treated wild type controls (Figure 2J). We observed an overall trend toward reduction of ipsilateral reactive astrogliosis of 27% across all 6 forebrain regions in the LPS-treated global properdin deletion group (Figure 2L) compared to LPS-treated wild type controls (Figure 2K) (main effect, *p* < 0.05, Kruskal-Wallis test), however the data did not reach significant values.

Additionally, assessment of microglial activation based on αM integrin immunoreactivity (Figure 2M) showed very low levels of αM+ microglia in saline-treated wild type controls (Figure 2N), and global properdin deletion did not affect those levels (Figure 2M). LPS-sensitization resulted in a substantial increase of microglial activation observed in the LPS-treated wild type group (Figure 2O) compared to the saline treated wild type controls (Figure 2M). Regional assessment shown in Figure 2M revealed a reduction in activation score of 31–66% in all 6 individual ipsilateral brain regions in the LPS-treated global properdin deletion group compared to the LPS-treated wild type controls (main effect, *p* < 0.05, Kruskal-Wallis test), however significance was reached only in hippocampus (Bonferroni correction, *p* < 0.05). At high magnification the αM+ cells in the LPS-treated wild type control group showed phagocytic morphology and high density (Figure 2O—insert, ipsilateral hippocampus) compared to the ramified resting phenotype and low density of these cells observed in the LPS-treated global properdin deletion animals (Figure 2P—insert, ipsilateral hippocampus).

## Discussion

Lack of oxygen to the fetal brain around the time of birth is a major cause of neonatal HI brain damage, triggering neurological sequelae such as cerebral palsy, epilepsy and mental retardation. Intrauterine or perinatal complications, including maternal infection, constitute a major risk for the development of neonatal HI brain damage. The mechanisms underlying the trigger of brain damage under the conditions of HI alone and LPS-sensitized HI overlap, but also differ (7, 29). Therefore, our study aimed to validate the effect of global properdin deletion in two models: HI alone and LPS-sensitized HI, thus addressing two different clinical scenarios.

In the current study, C57/Bl6 background was chosen as a result of the high severity of HI injury incurred following prolonged hypoxic exposure (7, 33).

Additionally, global properdin deletion reduced forebrain cell death and microglial activation, as well as tissue loss in a Rice-Vannucci model of neonatal HI and LPS-sensitized HI brain damage.

In the model of HI alone, deletion of properdin reduced brain damage based on evidence for TUNEL+ cell death and microglial post-HI response, which in both assessments reached significance.

Under the conditions of LPS-sensitized HI, properdin deletion reduced brain injury based on evidence of significantly diminished cell death, tissue loss and post-HI microglial activation. Overall, our data suggests a critical role for properdin, and possibly also a contribution in neonatal HI alone, as well as in infection-sensitized HI brain damage.

Complement is an essential part of innate immunity and participates not only in normal brain physiology, but also under pathological conditions, including ischemia (34). Absence of the CP in the neonatal HI brain is neuroprotective (35, 36). Despite the long history of research on the role of complement in neonatal HI (35, 36), there is a paucity of data on the participation of the AP in post-HI brain damage (34), in particular the role of properdin.

Our data shows that in HI alone and in LPS-sensitized HI, properdin deficiency reduced TUNEL+ cell death with significant differences in pyriform cortex, hippocampus, striatum, thalamus and overall in the HI alone set of experiments (Figure 1A), and in cortex, pyriform cortex and hippocampus in the LPS-sensitized HI (Figure 2A). Additionally, tissue loss was significantly reduced in the thalamus in the HI alone set of experiments (Figure 1D) and in the cortex and pyriform cortex in the LPS-sensitized HI (Figure 2B). Sub-regional differences in the vulnerability of the different brain regions to HI damage exist based on metabolic rate and energy demand (6). HI insult around term, as modeled in this study, damages predominantly gray matter in the cortex, hippocampus and/or thalamus (37). In animal models, damage in the cortex, thalamus and striatum has been associated with sensorimotor impairment (38–41). Interestingly, as the hippocampus is one of the regions with the highest metabolic rate in the developing brain and therefore highly susceptible to HI injury, damage to it and to the cortico-hippocampal projections causes memory and spatial processing dysfunction (42). Additionally, HI-induced reduction in hippocampal volume has been associated with impaired long-term reference memory, short-term working memory (43), as well as spatial navigation and recollection (44, 45). We observed reduced cell death in striatum of animals with global properdin deletion following neonatal HI (Figure 1A). As damage to the striatum, in particular to nucleus accumbens may have an impact on non-spatial navigation and learning (45, 46) and might explain non-spatial memory deficits in neonatal HI rats (37), protection of that region is essential. Neonatal HI is considered a major risk factor for psychiatric diseases including attention-deficit hyperactivity disorder (ADHD), autism, psychosis and schizophrenia (47–51). The main regions associated with related cognitive functions are the hippocampus and striatum as well as cortico-hippocampal and cortico-striatal projections. As global properdin deletion provides neuroprotection for these regions, it is likely that it would reduce the risk of development of later life psychological and behavioral complications, however that would require additional long-term behavioral studies.

Although there is no data on the effect of global properdin deletion on neonatal HI brain damage, our results are in line with previous studies looking at the role of the AP in murine models of stroke. In an adult mouse study of middle cerebral artery occlusion (MCAO), C3 deficiency and site-targeted inhibition with either CR2-Crry (inhibiting all pathways) or CR2-fH (inhibiting AP) significantly reduced infarct size, reduced apoptotic cell death, and improved neurological deficit score in the acute phase after stroke, but only CR2-fH provided sustained protection with no further development of injury in the subacute phase (52). Similarly, Ten et al. (36) demonstrated that C3 deficiency provided protection against MCAO as well as against neonatal HI. Additionally, intranasal C3a treatment ameliorated cognitive impairment in a mouse model of HI brain injury (35). However, C3 deficiency takes away the component central to all three complement pathways compared to properdin deficiency, which reduces these activities. Similarly, factor B-deficiency or CR2-fH treatment improved neurological function and reduced cerebral infarct, demyelination, P-selectin expression and neutrophil infiltration following MCAO in adult mice (52). Although the model of injury in MCAO is technically different than neonatal HI, both models share similarities, including oxygen deprivation and reperfusion, thus effects observed in MCAO could be plausible in neonatal HI and vice versa.

Our results show reduced microglial activation in all studied regions apart from cortex in the properdin deficient group following neonatal HI alone (Figure 1J). Similarly, in the LPS-sensitized HI model, we observed a main effect of the global properdin deletion with significant reduction observed in hippocampus (Figure 2M). This suggests reduced inflammatory response and subsequent cell death (Figures 1A, 2A). Inflammation plays a major part in the pathology of neonatal HI brain damage (6, 34, 53). Cerebral ischemia induces inflammation in both systemic circulation and the parenchyma. In an adult brain, this results in increased production of cytokines, as well as activation and migration of leukocytes to the injured brain (34, 53). In neonates, however, the result is an immediate innate immune response following the insult. The differences in the mechanisms between adult stroke and neonatal HI are mostly due to the immaturity of the neonatal CNS, resulting in insufficient ability to overcome excitotoxicity, oxidative stress and inflammation. HI damage is suggested to occur because of imbalance between pro- and anti-inflammatory cytokines, which boosts oligodendrocyte precursors to proliferate into astrocytes instead of oligodendrocytes, thus impairing subsequent myelination (54). In some models, presence of properdin has been associated with increased production of pro-inflammatory cytokines (TNF-alpha, IL-1b, and IL-6) and suppressed levels of anti-inflammatory cytokines (IL-10 and TGFb) (55). Therefore, its presence following HI might be a contributing factor for the imbalance between pro- and anti-inflammatory cytokines. We have previously shown that inhibition of IL-6 downstream products such as phosphorylated STAT3 is neuroprotective in neonatal HI (27). Hence, the lack of properdin would prevent IL-6 upregulation and provide neuroprotection in neonatal HI. Therefore, it can be assumed that deletion of properdin would exhibit a neuroprotective effect through reduction of pro-inflammatory cytokine levels, thus preserving the equilibrium between pro- and anti-inflammatory cytokines and ensuring subsequent myelination. Additionally, properdin is required for the AP activation when LPS is present (56). Thus, deficiency in properdin in the presence of LPS would prevent AP activation and ensuing inflammatory response. In addition to the increase of pro-inflammatory cytokines triggered by HI alone, LPS causes further upregulation of TNF-alpha, IL-1b, and IL-6. As properdin deletion might interfere with the execution of IL-6 dependent inflammatory response, it is possible that inherited properdin deficiency inhibits LPS sensitivity in neonates. Conversely, in a study looking at zymosan-induced and LPS-induced septic shock in adult mice, properdin deletion provides protection only in the case of zymosan-, but not in LPS-induced septic shock (57). However, the model of septic shock involves different mechanisms which can explain the differences in the effects of global properdin deletion.

Our data did not support an effect of properdin deletion on astroglial activation in HI alone or in LPS-sensitized HI, suggesting that the protective role of properdin in both models is likely due to impairment of the microglia-dependent pro-inflammatory response post-HI.

As a conclusion, our study provides evidence that properdin is involved and likely plays a key role in the trigger of neonatal HI and LPS-sensitized HI brain damage. Although our study was limited to male gender and the HI insult in the HI alone set of experiments was moderate rather than severe, global properdin deletion provides neuroprotection in the short term (48 h) in both models on the grounds of reduced cell death, tissue loss and microglial activation. The likely mechanism underlying these protective effects is impairment of the microglial pro-inflammatory response, which would prevent imbalance between pro- and anti-inflammatory cytokines following HI insult and would preserve subsequent myelination, however that would require further investigation. Overall our data suggest properdin as a novel target for treatment in neonatal HI brain damage; however, a better understanding of the pathway(s) through which it is involved in HI-brain damage would considerably improve the therapeutic potential of interfering with it in a clinical setting.

## Data Availability Statement

All datasets generated for this study are included in the article/supplementary material.

## Ethics Statement

This study was carried out in accordance with the UK Animals (Scientific Procedures) Act 1986 and the ARRIVE guidelines. The protocols were approved by the Home Office (PPL70/8784) and UCL Animal Welfare and Ethical Review Body.

## Author Contributions

CSi: collection and processing of data, writing, and editing the manuscript. QA-S, BS, and AC: collection and processing of data. CSt: provision of the properdin deficient animals, independent genotyping, writing, and editing the manuscript. MH: design of the study, processing of data, writing, and editing the manuscript.

### Conflict of Interest

The authors declare that the research was conducted in the absence of any commercial or financial relationships that could be construed as a potential conflict of interest.
